# Supplementary material for: Predicting visual function by interpreting a neuronal wiring diagram
Source: Nature. 2024 Oct 2;634(8032):113–23. doi: 10.1038/s41586-024-07953-5 (PMC11446822; doi:10.1038/s41586-024-07953-5)
Supplement: Supplementary file 6 — CRF and ERF predictions for individual TmY4 and TmY9 cells. Analogous to Supplementary Data 3, but for TmY target types. Shown are the top four monosynaptic pathways, the strongest pathway passing through each of the top ten intermediary types (ranking from Extended Data Fig. 7), and the trisynaptic pathway Tm1–TmY–Dm3–TmY (see the section entitled Prediction of spatial normalization). [file 41586_2024_7953_MOESM6_ESM.zip › DataS4/TmY9q/720575940605079724.pdf]

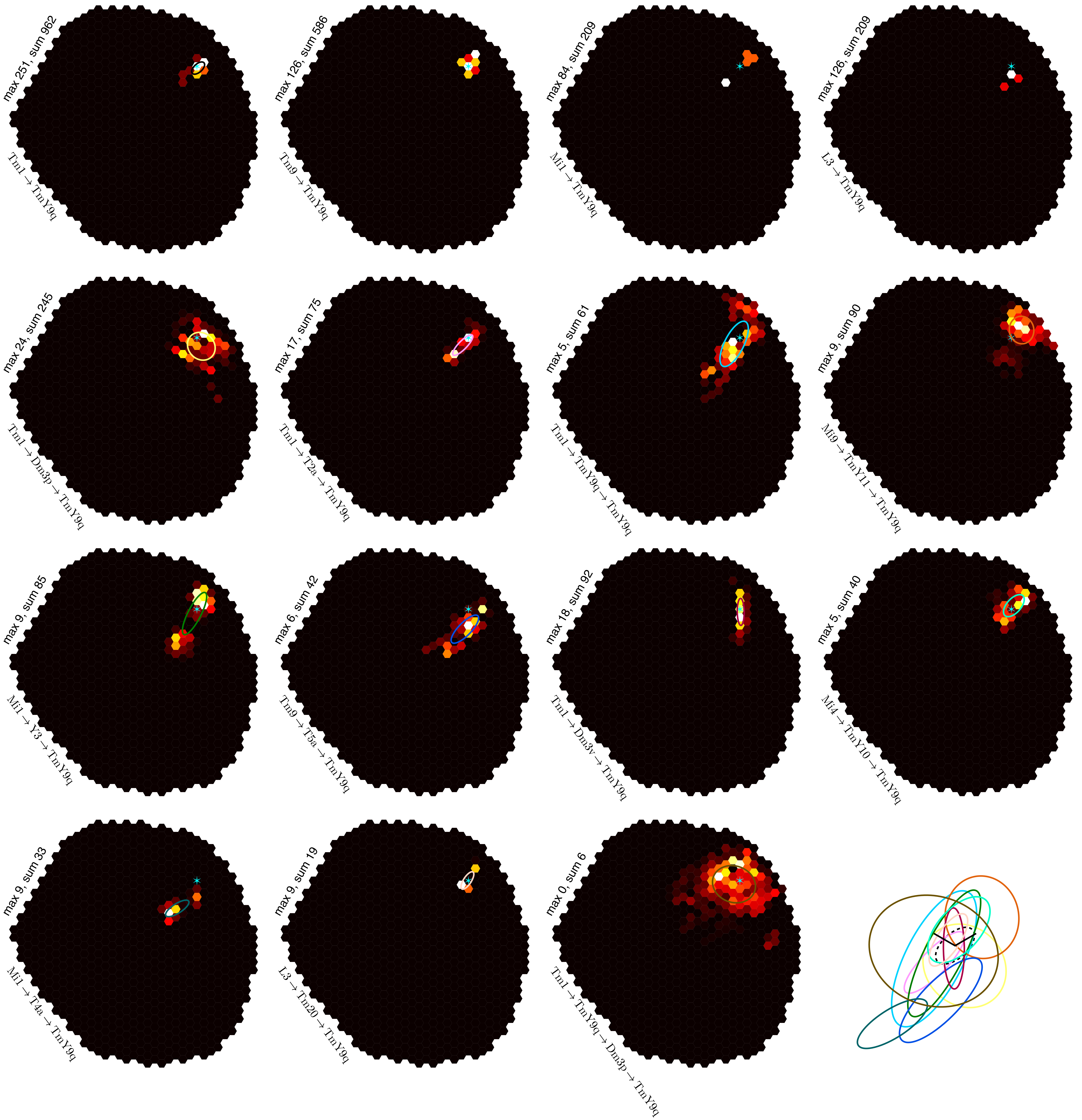
max 251, sum 962
Tm1 → TmY9q
max 126, sum 586
Tm9 → TmY9q
max 84, sum 209
Mi1 → TmY9q
max 126, sum 209
L3 → TmY9q
max 24, sum 245
Tm1 → Dm3p → TmY9q
max 17, sum 75
Tm1 → T2a → TmY9q
max 5, sum 61
Tm1 → TmY9q → TmY9q
max 9, sum 90
Mi9 → TmY11 → TmY9q
max 9, sum 85
Mi1 → Y3 → TmY9q
max 6, sum 42
Tm9 → T5a → TmY9q
max 18, sum 92
Tm1 → Dm3v → TmY9q
max 5, sum 40
Mi4 → TmY10 → TmY9q
max 9, sum 33
Mi1 → T4a → TmY9q
max 9, sum 19
L3 → Tm20 → TmY9q
max 0, sum 6
Tm1 → TmY9q → Dm3p → TmY9q
